# Paleodistribution modeling suggests glacial refugia in Scandinavia and out‐of‐Tibet range expansion of the Arctic fox

**DOI:** 10.1002/ece3.1859

**Published:** 2015-12-15

**Authors:** Marcelo Fuentes‐Hurtado, Anouschka R. Hof, Roland Jansson

**Affiliations:** ^1^ Departamento de Ecosistemas y Medio AmbientePontificia Universidad Católica de ChileSantiagoChile; ^2^ Landscape Ecology GroupDepartment of Ecology and Environmental ScienceUmeå UniversityUmeåSweden; ^3^ Department of WildlifeFish and Environmental StudiesSwedish University of Agricultural Sciences (SLU)UmeåSweden

**Keywords:** Arctic fox, ecological niche modeling, Fennoscandia, last glacial maximum, Out‐of‐Tibet hypothesis, refugia

## Abstract

Quaternary glacial cycles have shaped the geographic distributions and evolution of numerous species in the Arctic. Ancient DNA suggests that the Arctic fox went extinct in Europe at the end of the Pleistocene and that Scandinavia was subsequently recolonized from Siberia, indicating inability to track its habitat through space as climate changed. Using ecological niche modeling, we found that climatically suitable conditions for Arctic fox were found in Scandinavia both during the last glacial maximum (LGM) and the mid‐Holocene. Our results are supported by fossil occurrences from the last glacial. Furthermore, the model projection for the LGM, validated with fossil records, suggested an approximate distance of 2000 km between suitable Arctic conditions and the Tibetan Plateau well within the dispersal distance of the species, supporting the recently proposed hypothesis of range expansion from an origin on the Tibetan Plateau to the rest of Eurasia. The fact that the Arctic fox disappeared from Scandinavia despite suitable conditions suggests that extant populations may be more sensitive to climate change than previously thought.

## Introduction

The Quaternary has been a period characterized by a long‐term decrease in global temperature with recurrent oscillations between glacial and interglacial periods, associated with changes in global sea levels and the waxing and waning of ice sheets (Hewitt 2000). These changes have shaped the geographic distributions of plants and animal species (Schmitt 2007; Sérsic et al. 2011; Fraser et al. 2012). The last glacial maximum (LGM; about 21 kyr ago) has substantially influenced extant geographic distributions of species inhabiting high‐latitude regions (Martinez et al. 2004; Normand et al. 2011), and it is also representative of the recurrent cold periods during the last million years. Responses of species to glacial periods include range contractions and local extinctions (Dalén et al. 2007; Schmitt 2007; Stewart et al. 2010), as well as failure to recolonize previously glaciated areas due to dispersal limitation and biotic interactions (Normand et al. 2011).

The recently proposed “Out‐of‐Tibet” hypothesis of range expansion has suggested that many cold‐tolerant species originated in the Himalayan–Tibetan Plateau, the so‐called third pole, during the late Miocene and early Pliocene (3.60–5.09 Myr ago). They were subsequently favored by the cooling climate of the Quaternary and were able to colonize new areas (Deng et al. 2011; Tseng et al. 2014). One of these species, the Arctic fox (*Vulpes lagopus*), has been suggested to be the sister species to *Vulpes qiuzhudingi* living in Tibet during the Early Pliocene, implying that the Artic fox also originated in this area (Wang et al. 2014) and expanded its range during the Quaternary. Phylogenetic data have suggested that the Arctic fox' ancestor diverged from its sister species around 0.9 Ma ago (Perini et al. 2010) and then colonized Europe and North America during the mid‐late Pleistocene (Angerbjörn et al. 2004).

Since the Arctic fox is adapted physically and physiologically to cold conditions (Prestrud 1991; Audet et al. 2002; Wang et al. 2014), and given that remains have been found in different Pleistocene deposits over most of Europe and large parts of Siberia (Chesemore 1975), it is expected that it had a wide distribution during cold stages of the Pleistocene. However, the predicted geographic range of the Arctic fox during the LGM or other cold period has not been modeled, and the magnitude of range contraction since the LGM is hence poorly understood. Fossil data show that the species was present in mid‐latitude Europe during the last glacial (Dalén et al. 2007), but presently, it is only found in high northern tundra regions, and its Fennoscandian range is fragmented into several small isolated populations (Dalén et al. 2006; Norén et al. 2011). Moreover, ecological niche modeling under future climate change scenarios has shown that its southern range limit will likely move northwards and its range will contract in Fennoscandia, making populations in this area even smaller and more isolated (Hof et al. 2012). Extinction risk at the southern range limit of the arctic fox is exacerbated by the red fox (*Vulpes vulpes*), a species favored by human presence (Selås et al. 2010). It is thought that increasing and continued habitat loss of the Arctic fox due to competition with the red fox is the main driver of the Arctic fox's poor status in Fennoscandia (Hersteinsson and MacDonald 1992; Tannerfeldt et al. 2002; Selås and Vik 2006). Additionally, reduced availability of the preferred food item of the Fennoscandian arctic fox population, the Norway lemming (*Lemmus lemmus*) may form another threat in the future (Hof et al. 2012). Modeling how the Arctic fox responded to previous climatic events may improve predictions of how Arctic fox populations, and other cold‐adapted species, will respond to a warming climate.

A recent phylogeographic study by Dalén et al. (Dalén et al. 2007) suggested that the Arctic fox went extinct in mid‐latitude Europe at the end of the Pleistocene and did not track its climatic niche when it shifted northwards. Instead, high genetic similarity between the extant populations in Scandinavia and Siberia suggests an eastern origin for the postglacial Scandinavian populations (Dalén et al. 2007), and that the European populations were unable to track their habitat in response to climate change. However, some evidence suggest that the Arctic fox was present in Fennoscandia during the last glacial. Frafjord and Hurthammer (Frafjord and Hufthammer 1994) described 44 naturally deposited Arctic fox bones from the Norwegian coastline dated to 36,000–28,000 and 13,000 B.P. Furthermore, a mitochondrial DNA (mtDNA)‐based molecular study (Fedorov and Stenseth 2001) showed that the Norway lemming was able to survive in glacial refugia in Fennoscandia, which, given that it is the main food source of the Fennoscandian Arctic fox population (Kaikusalo and Angerbjörn 1995; Dalerum and Angerbjörn 2000; Elmhagen et al. 2000), implies that the Arctic fox may also have been able to survive there. If so, it might be that the Fennoscandian population went extinct during the warmest period in the Holocene, followed by recolonization from the east or that the extinction hypothesis based on molecular data should be further explored: Phylogeographical hypotheses should be evaluated spatially to test the scope of their conclusions (Peterson and Lieberman 2012; Peterson et al. 2012). This is especially important if they have implications for management and conservation decisions. In this context, elucidating the response of the Arctic fox to past climate changes will not only contribute to the biogeographic knowledge of the region, but also inform about the sensitivity of the critically endangered Fennoscandian Arctic fox population (Angerbjörn et al. 2008) to warming of the climate, and help improving current conservation strategies.

Here, we use ecological niche modeling (ENM) to identify the climatic niche of the Arctic fox under current environmental conditions to enable predicting the suitable climatic conditions for the species during the last interglacial (LIG), LGM (a cold stage) and the mid‐Holocene (a warm stage). First, we modeled the geographic range of the Arctic fox during the LGM and the mid‐Holocene (about 6000 years ago) to provide the climatic context to its demise in Europe and putative recolonization from the east. Second, we explore the “bioclimatic” connectivity of the Himalayan–Tibetan Plateau with high‐latitude circumpolar areas inhabited by the Arctic fox during the LIG and LGM to evaluate the “Out‐of‐Tibet” hypothesis, with LIG and LGM conditions taken to be representative for nonglacial and glacial periods since the mid‐Quaternary origin of the species (Perini et al. 2010). These efforts help shed light on risks for population extinction and range contraction in response to future climate change.

## Methods

**Figure 1:**
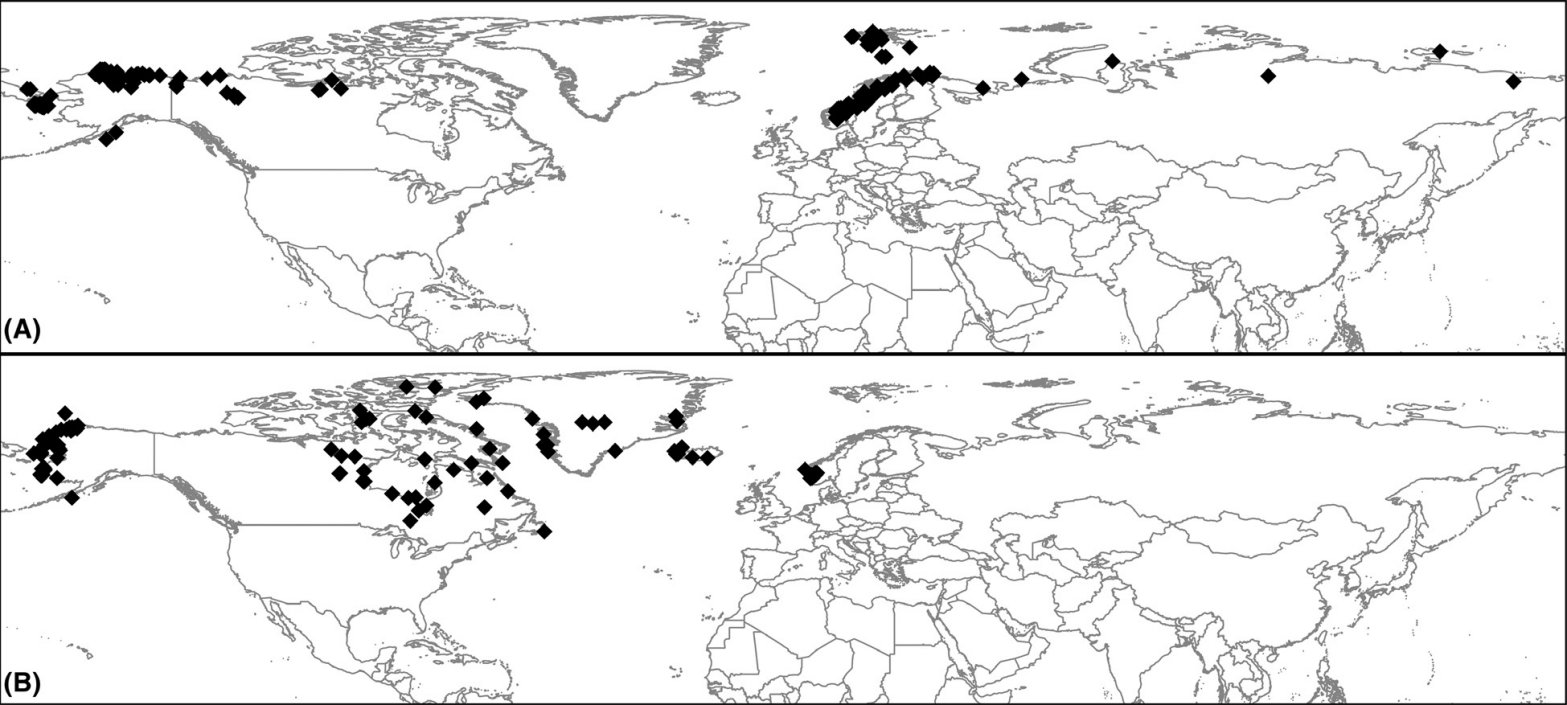
A map of the study area used to build the models with model calibration dataset (A) and model evaluation dataset (B).

We used the ENM algorithm MaxEnt version 3.3.3.k (Phillips et al. 2006) to predict the potential current and past suitability of the Arctic fox, because it has shown to be a good approach and widely used algorithm to assess species' ecological niche when absence data is lacking (Elith et al. 2006; Hijmans and Graham 2006). Ecological niche models are frequently used to predict the impact of climate change on the geographic distribution of suitable areas of species, both for projecting responses to future climate change and for hindcasting effects of past climate events (Carnaval and Moritz 2008; Virkkala et al. 2010; Araújo et al. 2011). The general approach is to identify the variables that determine most of the variation in species presence. Models subsequently predict the likelihood of species presence in a defined region at a specific time (Phillips et al. 2006). Maxent uses the algorithm of maximum entropy to find the probability distribution most close to the uniform, but with restriction of the observed environmental values (Elith et al. 2011). ENM outputs are strongly affected by the study area extent; therefore, the study area delimitation must consider biogeographic features reflecting the dispersal capacity of the species and the historical species distribution (Barve et al. 2011). However, as biogeographic barriers are dynamic rather than static, the study area was delimited to encompass all known historical and current occurrences of the species including all land areas inside (Hersteinsson and MacDonald 1992; Dalén et al. 2007; Perini et al. 2010; Wang et al. 2014) (Fig. 1).

Species occurrences were collected from the public online data source the Global Biodiversity Information Facility (GBIF, http://www.gbif.org/). Only the observations that fell within the geographic range of the species published by the IUCN Red List of Threatened Species were taken into account in order to omit observations of, for instance, individuals escaped from fur farms. To reduce sampling bias caused by spatially clustered occurrences and to reduce model overfitting resulting from duplicate locations, occurrences were filtered to obtain one occurrence per pixel of the environmental layers (~5 km at the equator). We also used a distance filter of 50 km between occurrences to further explore the effect of sampling bias in our results. To assess the accuracy of the models, we split the occurrence datasets in two subsets, one set for calibration and another for evaluation (Fig. 1). To avoid environmental dependency between subsets for this cross‐validation approach, these were divided geographically by longitude.

To calibrate the model of the current ecological niche of the Arctic fox, bioclimatic data for the time period 1950 – 2000 at the 2.5 arc‐minute scale (~5 km at the equator) were used (Hijmans et al. 2005). The 19 bioclimatic variables (see Table S1) used in the models were derived from monthly temperature and rainfall values and are available at WorldClim (http://worldclim.org/bioclim). To explore the effect of overfitting, we included a variable reduction step based on a correlation matrix, a jackknife analysis and evaluation of environmentally meaningful variables for the species. In addition, in a second run of the models we, instead of the variable reduction step, took advantage of the regularization application of MaxEnt. This application deals with the selection of environmental variables (regulating some to zero) and prevents MaxEnt from matching the input data too closely. This may lead to overfitting of the data, which has a negative effect on the predictive performance of models (Phillips and Dudík 2008). This application has shown to perform well and is thought to outperform preselecting procedures like PCA (Hastie et al. 2009; Elith et al. 2011). For the LGM and mid‐Holocene model projections, we used general circulation model (GCM) simulations from two climate models: the Community Climate System Model (CCSM4) (Gent et al. 2011) and the Model for Interdisciplinary Research on Climate (MIROC‐ESM 2010) (Watanabe et al. 2011). The LGM climate over Europe as simulated by CCSM is colder and drier than that of MIROC (Schorr et al. 2012) and allowed us to evaluate modeling performance with two sets of climate data. For the LIG data model projection, we used the only data set available (Otto‐Bliesner et al. 2006).

One hundred models were performed for both approaches dealing with overfitting using the bootstrap replication method in MaxEnt. We used the medians of the replicates as outputs. To convert the continuous output into binary maps, continuous values were extracted using the calibration occurrences. The binary threshold was set above the lowest 10 percentile of the calibration occurrences, assuming that those lowest values would represent error in identification of georeferencing, individuals in sink populations, migrating or juvenile individuals looking for nonoptimum habitats (Pineda and Lobo 2012). The evaluation areas were sampled with the evaluation dataset to assess whether the occurrences were predicted correctly. To estimate the capacity of the models to correctly predict occurrences of species, we followed recommendations by Peterson et al. (2012). We calculated cumulative binomial probability distributions, in which we used the predicted points of the validation as measures of success, the proportion of area predicted to be suitable as a null expectation of the probability of success, and the total number of evaluation occurrences as the number of trials, estimating a *P*‐value. Once the significance of the models was evaluated, we used all occurrences to provide more information during the calibration of the final models and to obtain the final thresholds that we applied to generate binary models. As we were interested in establishing the species potential suitable areas of occurrence for the species (Peterson et al. 2012) during the LGM, strict model transference methods were used also avoiding perilous extrapolation (i.e., clamping and extrapolation were turned off) (Owens et al. 2013). This technique allows model transference from the calibration area to explore novel environments in different areas or time periods. To evaluate model transferability, we used two approaches: the mobility‐oriented parity (MOP) and the multivariate environmental similarity surface (MESS) (Elith et al. 2010; Owens et al. 2013). Specific parameters were a sample of 0.5% of the entire cells available in the raster layers of the study area (i.e., 4.8 × 10^6^ for current and mid‐Holocene raster layers and 6.3x10^6^ for LGM) and 50% cells of current climate raster layers to compare with the past climate variables. Key variables compared were minimum and maximum temperature and precipitation in the driest month. Model transference allowed us to project the species ENM to environmental scenarios in the LIG, LGM, and mid‐Holocene.

For a further evaluation of the LGM predictions, Eurasian Late Pleistocene occurrences of the Arctic fox, downloaded from the New and Old Worlds (NOW) database of fossil mammals (Fortelius 2013) on 7 November 2014, were used. We then evaluated whether our LGM models of the geographic range of the Arctic fox predicted presence at the fossil localities. To evaluate the contribution of each of the 19 bioclimatic variables in the Maxent models, response curves and jackknife analyses were performed. Finally, the gap between the European easternmost area of suitable habitat and the suitable areas on the Tibetan–Himalayan plateau was calculated for the LIG, LGM, mid‐Holocene, and present.

## Results

Below we present the results using filtering of the occurrence data to obtain one occurrence per pixel and using the regularization application of Maxent to control for overfitting. The models based upon these approaches performed better than the models generated after applying a distance filter between occurrences and applying a variable reduction step. The results generated with the latter methodology are presented in the Appendix.

**Figure 2:**
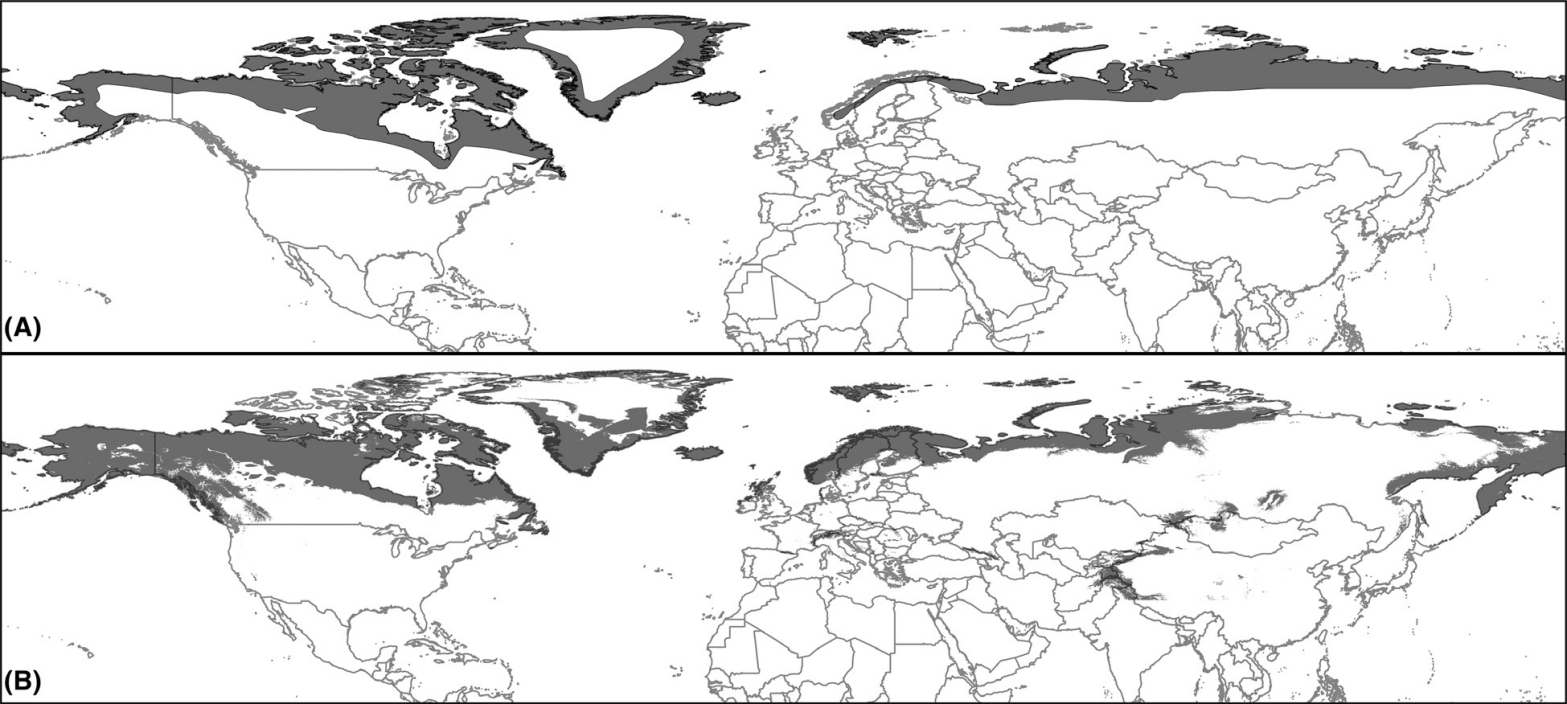
Map of the study area with IUCN species distribution (A) and with predicted present distribution obtained from models (B).

We obtained 347 Arctic fox occurrences in total (Fig. 1). Using the calibration dataset (60% of 347 occurrences) over the median output, we obtained a 10 lowest percentile threshold of 0.041. This threshold was applied to acquire the binary predictive map of the present conditions, which was used for model evaluation. A total of 124 occurrences were predicted correctly of 139 validation occurrences (40% of 347 occurrences), which equates to an omission error of 11%. In total, 13% of the study area was predicted as suitable, obtaining a significant probability of prediction of the evaluation occurrences (*P *<* *0.0001). ENMs for present conditions constructed with the complete dataset of occurrences were obtained and converted to binary maps using a new 10 lowest percentile threshold of 0.08 based on the complete dataset. This threshold was applied to the LIG, LGM, and mid‐Holocene median outputs to obtain the final maps (Fig. 2).

MESS analysis showed broad areas with high (red) and low (blue) environmental similarity between current and past climate conditions (see Fig. S1). MOP analyses revealed sites where nonanalogous environmental conditions were found (black). Nonanalogous climates appeared in central North America, central areas of Greenland and in Russia. We failed to find nonanalogous climates for the mid‐Holocene. Thus, as we used Maxent with strict model transference (i.e., no extrapolation), model outputs only show suitable areas available in past climate.

**Figure 3:**
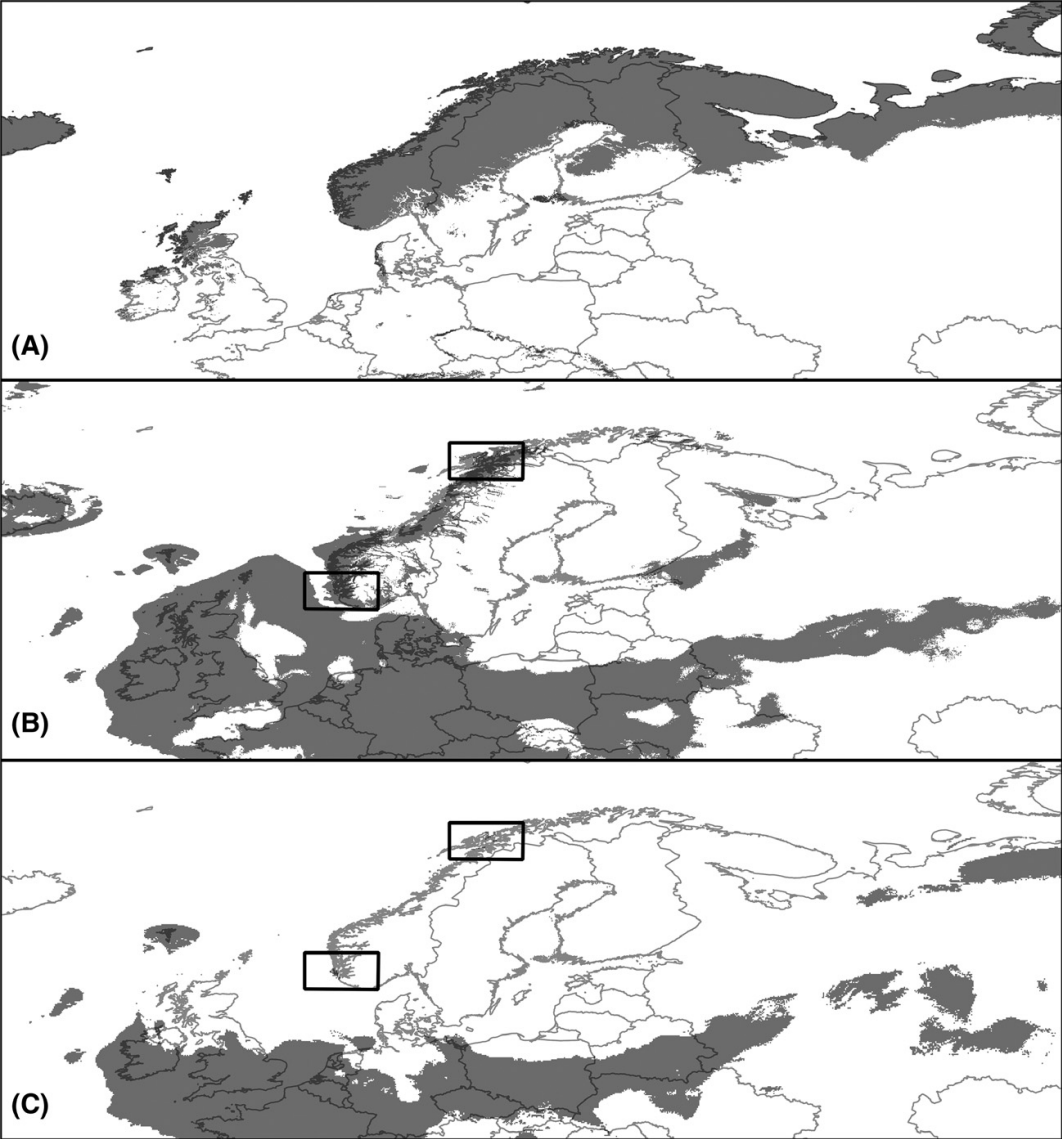
Ecological niche modeling results for Fennoscandia (A) Present; (B) CCSM‐4 based LGM, (C) MIROC‐ESM based LGM. Squares: areas predicted as suitable refugia in Norway in the MIROC‐ESM LGM ENM.

Projection of the species niche to LGM climate indicated refugia areas in close proximity to the ice sheets along the coast of Norway as well as in many suitable areas in Central Europe, Siberia, and Asia, with a greater geographic range of the species than in the present (Fig. 3). The CCSM‐4 paleoclimatic models predicted more area to be suitable than the MIROC‐ESM models. The MIROC‐ESM model projection showed that only two small areas along the coast of Norway might have been suitable as glacial refugia. Despite these differences, the two paleoclimatic models were largely similar for the LGM (Fig. 3).

**Figure 4:**
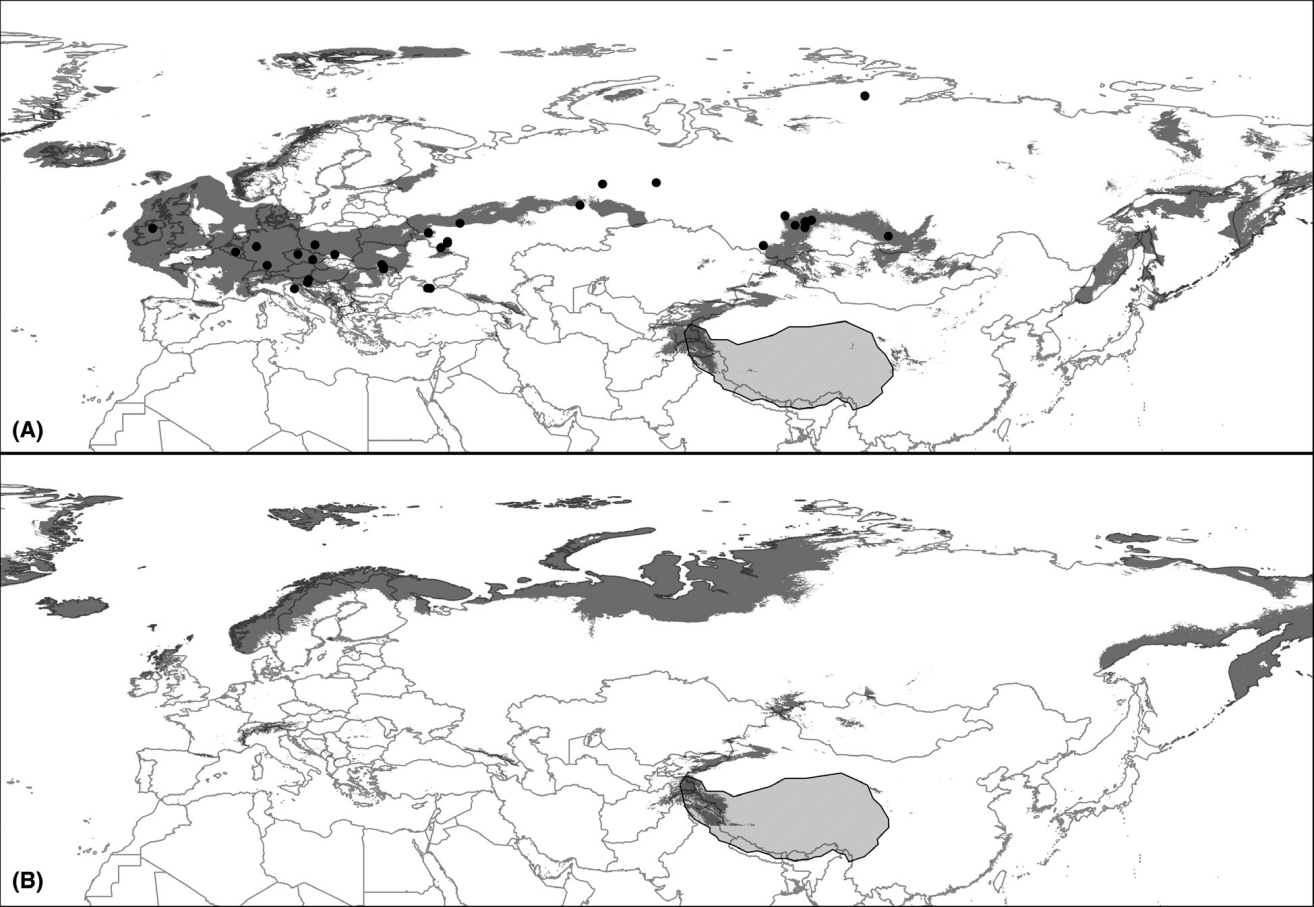
Ecological niche modeling results for Eurasia during mid‐Holocene and LGM based in the CCSM‐4 model. (A) Last Glacial Maximum ENM; (B) Mid‐Holocene ENM. Light gray area: Himalayan–Tibetan Plateau. Black dots: Arctic Fox Pleistocene fossil records over LGM ENM.

The locations of late Pleistocene Arctic fox fossils obtained from the NOW database were predicted as areas of presence in 69% and 56% of the cases, using the LGM predictions based on the CCSM‐4 models and MIROC‐ESM models, respectively. The models also predicted habitat suitability in Tibet and many parts connecting Tibet and the known LGM range of the species, with a minimum distribution gap of 2000 km (Fig. 4). Suitable areas in the Tibetan–Himalayan plateau were predicted for all climate scenarios. Projection of the species niche to the warmer climates during the LIG and mid‐Holocene showed a substantial range contraction and northwards shift compared to the LGM range, but the models still predicted presence of the species in Fennoscandia and the Tibetan–Himalayan plateau (Fig. 4).

**Figure 5:**
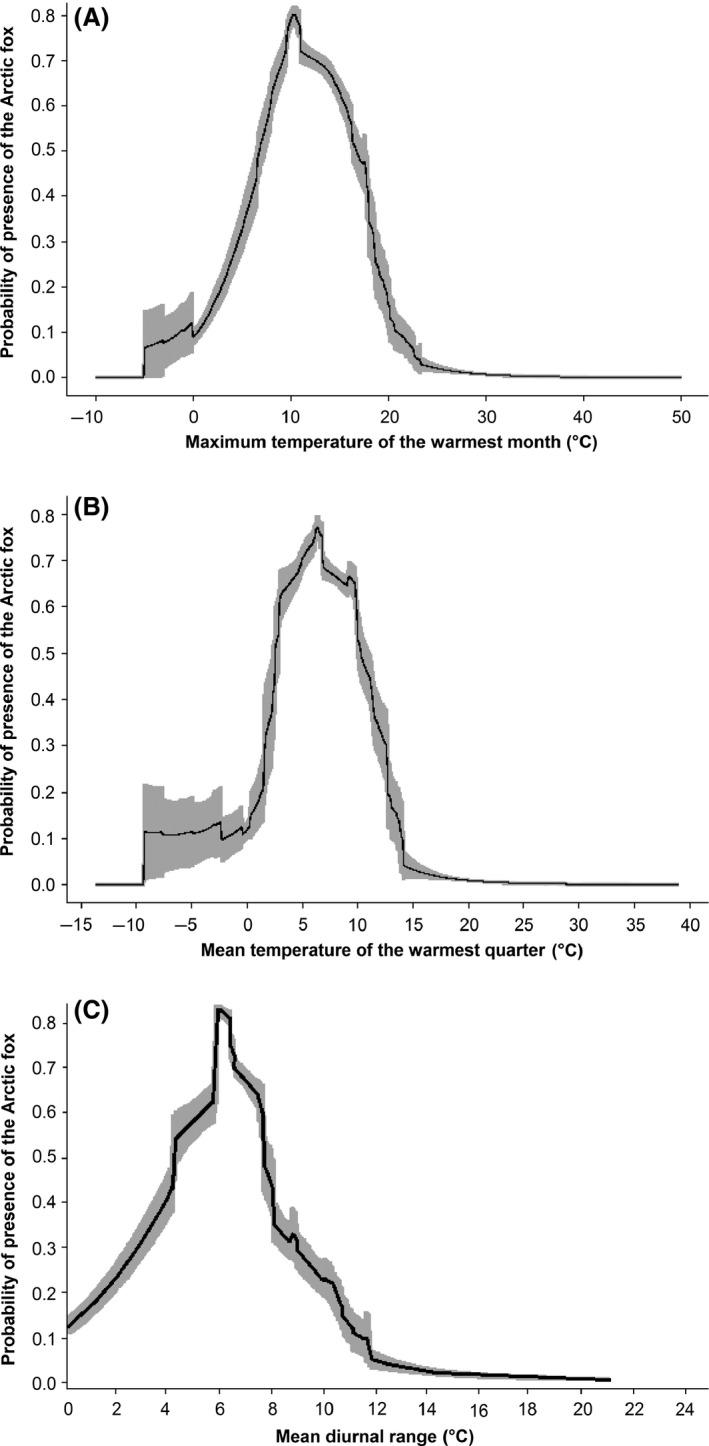
Environmental variables contribution to model predictive power. (A) Maximum temperature of the warmest month (BIO5), (B) Mean temperature of the warmest quarter of the year (BIO10) and (C) Mean diurnal range (the mean of the monthly maximum temperature minus the minimum temperature, BIO2).

Through jackknife evaluation of the importance of the environmental variables to the model, we found that the maximum temperature of the warmest month, the mean temperature of the warmest quarter of the year, and the mean diurnal range (the mean of the monthly maximum temperature minus the minimum temperature) were the most relevant to infer species presence (Fig. 5). When evaluating the response curves of each of these variables, we observed that the Arctic fox is not likely to occur in areas where the maximum temperature of the warmest month is below approximately −5°C or above 25°C. Also, when the mean temperature of the warmest quarter of the year is either below −10°C or above 15°C the area is not likely to be suitable for the Arctic fox. Furthermore, when the mean difference between monthly maximum and minimum temperatures exceeds 12°C, the area is not likely to be suitable.

## Discussion

Our paleodistribution models of potential suitable areas suggest that refugia for Arctic fox might have existed along the Norwegian coastline during the LGM, providing support for local survival in Fennoscandia during the last glacial. When applying the variable reduction approach, only the CCSM GCM models predicted the existence of glacial refugia along the Norwegian coastline. On the other hand, when relying on the regularization parameter of Maxent, both the MIROC‐ESM and the CCSM models predicted the existence of glacial refugia along the Norwegian coast. The approaches and GCM's slightly differed in the extension of suitable areas predicted for the Arctic fox in the rest of Europe. The fact that 1) the omission error was larger and 2) the percentage of fossil record locations predicted as suitable was lower when applying variable reduction than when relying on the regularization parameter, may indicate that the former approach were too restrictive. Irrespective of which approach is superior, glacial refugia along the Norwegian coastline were predicted twice under the CCSM GCM and once under the MIROC‐ESM model. Furthermore, modeling refugial areas rely on how well global circulation models estimate the LGM climate. The CCSM‐4 paleoclimatic models predicted more area to be suitable than the MIROC‐ESM models, and better predicted the Late Pleistocene fossil records of the species. In addition, previous evaluations of the performance of CCSM and MIROC concluded that MIROC performs well for areas near the equator, while CCSM performs better at high latitudes (Masson‐Delmotte et al. 2005), suggesting that predictions of the LGM distribution based on CCSM‐4 may be more reliable. Therefore, our model predictions and fossil records support the hypothesis of local survival in Norway.

The molecular evidence suggesting extinction of the Arctic fox living in mid‐latitude Europe during the LGM is mostly based on the use of mtDNA comparisons between Late Pleistocene arctic foxes populations in midlatitude Europe with those from extant Siberian and Scandinavian populations (Dalén et al. 2007). However, results should be interpreted cautiously considering that mtDNA represents a few genetic loci that might or not might reflect the overall history of the genome (Hofreiter et al. 2001). Although ancient DNA is a powerful tool, further explorations of populations dynamics related to glacial events (e. g. Dalén et al. 2007; Valdiosera et al. 2007) should also be explored using nuclear DNA sequences, morphological data (Hofreiter et al. 2001; Leonard 2008), and/or a habitat suitability approaches.

The LIG and mid‐Holocene projections revealed a pattern of habitat contraction during warmer periods, but with persistence of suitable areas in Fennoscandia. These mid‐Holocene predictions were consistent between the two GCMs used. Thus, according to our models, habitat loss during warm periods alone cannot explain the putative extinction of Pleistocene populations from Europe (Dalén et al. 2007). Moreover, the northwards habitat tracking needed for survival at the end of the last glacial would have been modest if Arctic fox were continuously present in Fennoscandia close to the ice sheet, as suggested by our models. A scenario that would reconcile the ENM and phylogeographic results is that the Arctic fox is more sensitive to range contraction and population declines than formerly believed. If so, the sensitivity of the critically endangered present Fennoscandian population to present and future climate change might also be higher than formerly believed.

To assess the scope of our results, an important factor to consider is the accuracy of the ENM methodology. We followed a methodology to obtain predictions with an associated *P*‐value during the calibration and evaluation processes (Peterson et al. 2012). This methodology allowed us to minimize spatial autocorrelation among occurrences. We further used two different approaches to minimize overfitting and applied a recent method to evaluate the predictive power of models following Peterson et al. 2012;. In addition, it is imperative to take fossil records into account in the evaluation process, when available. The finding of naturally deposited Arctic fox bones along the Norwegian coastline dated to 36,000–28,000 and 13,000 B.P. (Frafjord and Hufthammer 1994) strongly indicates that the species occupied the suitable areas in Fennoscandia during the last glacial predicted by our models. The use of the NOW database of Late Pleistocene fossil records of the Arctic fox in Europe also supported our LGM model. It is important to highlight that reactions to Pleistocene climatic change are species‐specific (Taberlet and Cheddadi 2002) and that generalizations should be made with caution, especially when considering conservation management (Schmitt 2007).

The LGM results suggest that there was approximately 2000 km between suitable conditions in Europe/western Asia and on the Tibetan–Himalayan Plateau. This is a relatively short distance for the Arctic fox, considering its capacity to travel long distances (Eberhardt and Hansson 1978), thus providing spatial support for the “Out‐of‐Tibet” hypothesis. In the context of the “third pole” to north pole colonization hypothesis, the high percentage of Pleistocene occurrences available (Fortelius 2013) that were predicted by the LGM ENM suggests both high predictive power of the models and that these regions were relatively well connected during cold stages of the Pleistocene. Mobility across land and sea ice has been described as an important factor for the geographic distribution of the species, maintaining connectivity among populations across its range (Norén et al. 2011; Mellows et al. 2012). Taken together, our results suggest that there may have been migration routes for the range expansion of the species from its putative area of origination, but potential connection routes should be further explored with more fossil and molecular data.

The present day geographic range of the Arctic fox as predicted by our models is slightly over‐extensive when comparing it to the IUCN map. This is likely caused by the fact that at present the distribution range of the Arctic fox is constrained by a multitude of factors, including human pressure (Faurby and Svenning 2015). One of the main limitations of ENM algorithms is that they assume that climate largely determines species' geographic ranges due to the limited possibility of introducing other biotic and abiotic factors in models. Species interactions may be accounted for in ENMs, and such interactions have in fact been used to study the impact of future climate change on the Arctic fox (Hof et al. 2012). In Hof et al.'s study, the current and potential future range of the red fox (*Vulpes vulpes*), being a superior competitor to and predator of the Arctic fox, as well as the Norway lemming, the main food source of the Arctic fox, were incorporated in the niche model for the Arctic fox (Hof et al. 2012). This study showed that the current geographic range of the Arctic fox is partly constrained by the presence of the red fox. Incorporating the geographic range of the red fox into our models might have improved the prediction of the current distribution of the Arctic fox. However, as the red fox is not well‐adapted to cold climates and the northern limit of its distribution is correlated with climate‐related resource availability (Hersteinsson and MacDonald 1992), we argue that the red fox is unlikely to have affected the ability of the Arctic fox to survive in glacial refugia. We therefore did not incorporate the range of the red fox in our modeling. Yet, it might be that competition from the red fox caused the demise of relict Arctic fox populations in Fennoscandia during the Holocene, despite that our models suggested climatic suitability in the mid‐Holocene (6000 years BP).

Declines in Norway lemming distribution and abundance might also account for a Holocene extinction of the Arctic fox from Fennoscandia. As previously mentioned, it has been suggested that the Norway lemming was able to survive in glacial refugia in Fennoscandia (Fedorov and Stenseth 2001). Although the Arctic fox is generally classified as an opportunistic omnivore that can adapt its diet to the abundance of prey items (Stickney 1991; Dalerum and Angerbjörn 2000), the Arctic fox population in Fennoscandia belongs to a “lemming ecotype” specializing on lemmings (*Lemmus* sp.) (Kaikusalo and Angerbjörn 1995; Dalerum and Angerbjörn 2000; Elmhagen et al. 2000). The abundance of the Fennoscandian Arctic fox has in the past reflected the abundance of lemming species (Angerbjörn et al. 1995; Kaikusalo and Angerbjörn 1995) and the suggestion that the Norway lemming was able to survive in local refugia therefore gives weight to our findings that the Arctic fox may have been able to do so as well.

Knowledge derived from ENMs has been recognized as a research priority in conservation biogeography, given its contribution to assess conservation priorities, evolutionary patterns and climate change effects on biodiversity (Richardson 2012). In this context, we have demonstrated how the use of ENM to evaluate the paleodistribution of a species could be an informative tool, especially when combined with fossil records and ancient DNA evidence. This exercise allowed us to shed light on the complex evolutionary history of the Arctic fox to improve assessments of its sensitivity to future climate change.

## Data Accessibility

All Arctic fox occurrences are freely available in the Global Biodiversity Information Facility (GBIF, http://www.gbif.org/) and the New and Old Worlds (NOW) database of fossil mammals, University of Helsinki.

## Conflict of Interest

None declared.

## Supporting information


**Table S1**. Predictor variables used in the ecological niche models.
**Figure S1**. MOP and MESS model transference analyses.

**Appendix S1**. Alternative results including further occurrence and variable reduction steps.
