# Supplementary material for: Paleodistribution modeling suggests glacial refugia in Scandinavia and out‐of‐Tibet range expansion of the Arctic fox
Source: Ecol Evol. 2015 Dec 15;6(1):170–80. doi: 10.1002/ece3.1859 (PMC4716496; doi:10.1002/ece3.1859)
Supplement: Supplementary file 1 — Table S1. Predictor variables used in the ecological niche models. Figure S1. MOP and MESS model transference analyses. [file ECE3-6-170-s001.docx]

# Supplementary material

**Table S1:** Predictor variables used in the ecological niche models.

| Variables | Explanation |
| --- | --- |
| BIO 1 | Annual mean temperature |
| BIO 2 | Mean diurnal range (Mean of monthly (max temp - min temp))  (mean of monthly (maximum temperature - minimum temperature)) |
| BIO 3 | Isothermality (BIO2/BIO7 * 100) |
| BIO 4 | Temperature seasonality (standard deviation * 100) |
| BIO 5 | Maximum temperature of warmest month |
| BIO 6 | Minimum temperature of coldest month |
| BIO 7 | Temperature annual range (BIO5-BIO6) |
| BIO 8 | Mean temperature of wettest quarter |
| BIO 9 | Mean temperature of driest quarter |
| BIO 10 | Mean temperature of warmest quarter |
| BIO 11 | Mean temperature of coldest quarter |
| BIO 12 | Annual precipitation |
| BIO 13 | Precipitation of wettest month |
| BIO 14 | Precipitation of driest month |
| BIO 15 | Precipitation seasonality (coefficient of variation) |
| BIO 16 | Precipitation of wettest quarter |
| BIO 17 | Precipitation of driest quarter |
| BIO 18 | Precipitation of warmest quarter |
| BIO 19 | Precipitation of coldest quarter |

**Figure S1:** MOP and MESS model transference analyses.

**
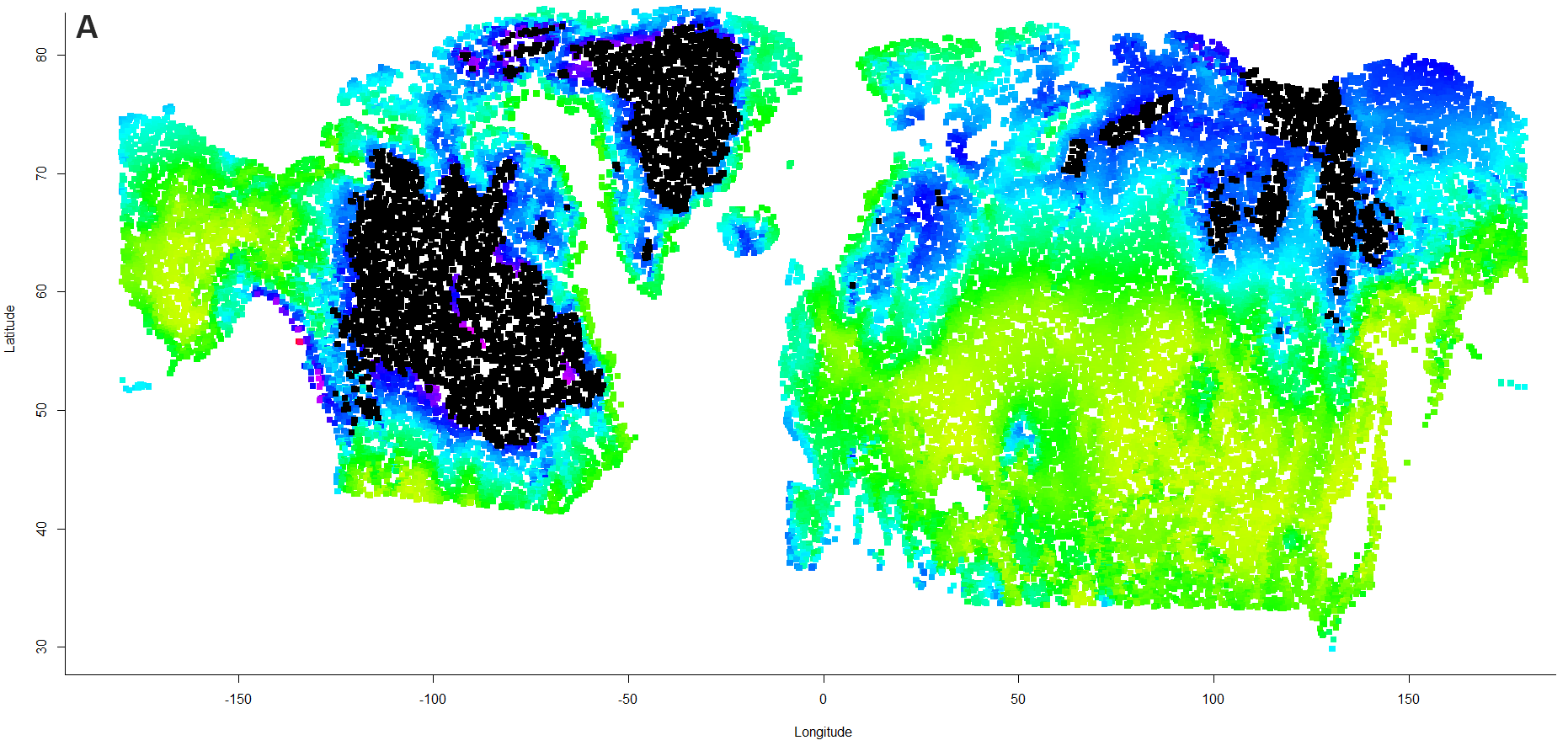

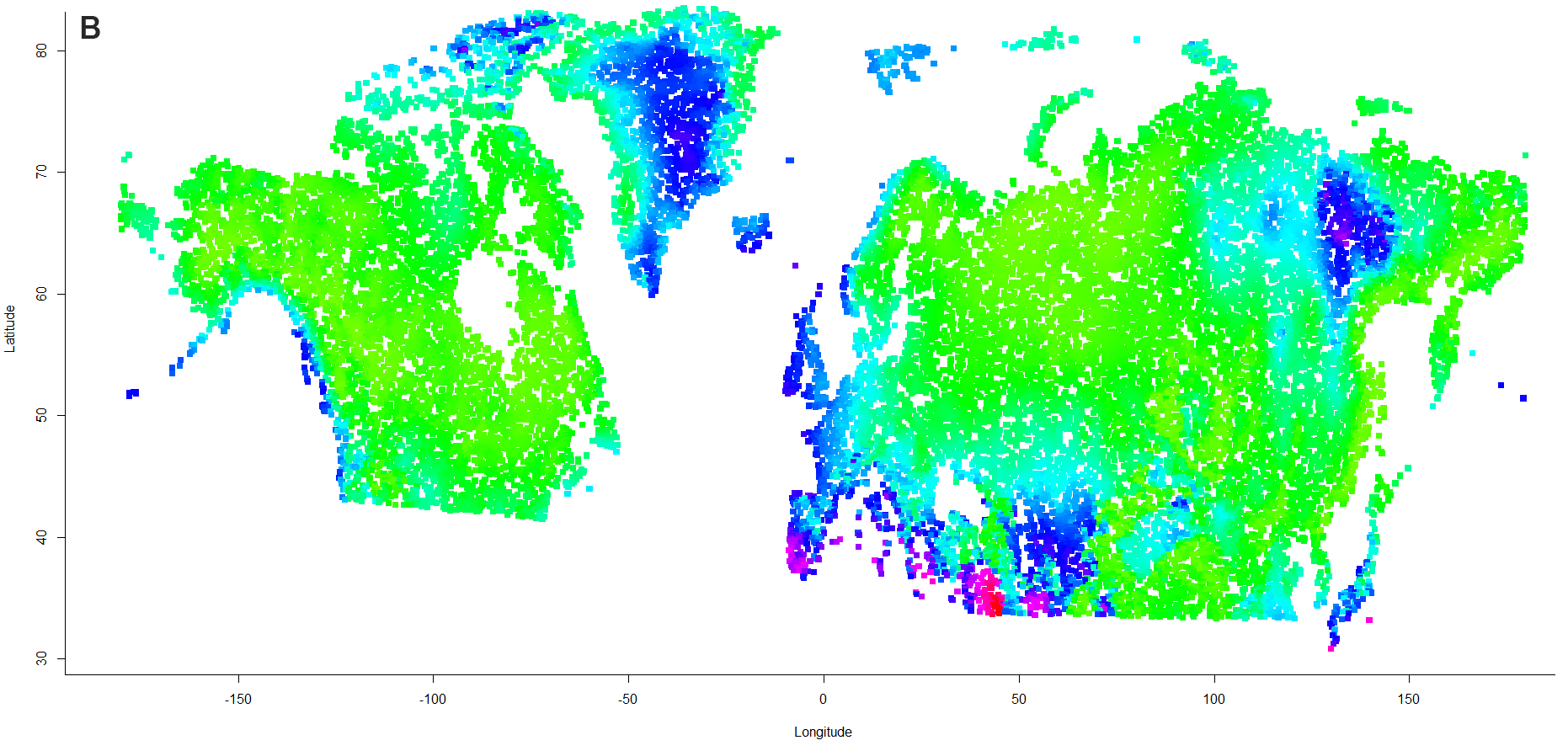
**

Analogous climate comparisons between current and past climate. Comparisons are shown in color range as areas of high (red) and low (blue) similarity with current climate conditions based on the MESS metric (Elith et al. 2010). Areas with a lack of analogous climates to current climate conditions were identified (black) based on MOP analyses (Owens et al. 2013).
