# Supplementary material for: Paleodistribution modeling suggests glacial refugia in Scandinavia and out‐of‐Tibet range expansion of the Arctic fox
Source: Ecol Evol. 2015 Dec 15;6(1):170–80. doi: 10.1002/ece3.1859 (PMC4716496; doi:10.1002/ece3.1859)
Supplement: Supplementary file 2 — Appendix S1. Alternative results including further occurrence and variable reduction steps. [file ECE3-6-170-s002.docx]

**Appendix**

Below we present the results using a distance filter between occurrences and applying a variable reduction step. Using this methodology we obtained 216 Arctic fox occurrences in total. Using the calibration dataset (60% of 216 occurrences) over the median output, we obtained a 10 lowest percentile threshold of 0.34. This threshold was applied to acquire the binary predictive map of the present conditions, which was used for model evaluation. A total of 52 occurrences were predicted correctly out of 86 validation occurrences (40% of 216 occurrences), which equates to an omission error of 17%. In total 14% of the study area was predicted as suitable, obtaining a significant probability of prediction of the evaluation occurrences (*p* < 0.0001). ENMs for present conditions constructed with the complete dataset of occurrences were obtained and converted to binary maps using a new 10 lowest percentile threshold of 0.20 based on the complete dataset. This threshold was applied to the LIG, LGM, Mid-Holocene median outputs to obtain the final maps.

Projection of the species niche to LGM climate indicated refugia areas in close proximity to the ice sheets along the coast of Norway as well as in many suitable areas in Central Europe, Siberia and Asia, with a greater range size of the species than in the present. The MIROC-ESM model projection showed wide niche suitability in Europe but no refugia areas in Scandinavia, only CCSM-4 paleoclimatic models predicted refugia in Scandinavia. Despite these differences, the two-paleoclimatic models were largely similar for the LGM. The locations of late-Pleistocene Arctic fox fossils obtained from the NOW database were predicted as areas of presence in 38% and 25% of the cases when using the variable reduction approach, using the LGM predictions based on the CCSM-4 model and MIROC-ESM models, respectively.
